# Association Between Hearing Loss and Systemic Small-Vessel Vasculitis: Audiological Aspects Across Disease Types

**DOI:** 10.3390/medicina61071117

**Published:** 2025-06-20

**Authors:** Vija Vainutienė, Eugenijus Lesinskas, Tatjana Ivaškienė, Diana Mieliauskaitė, Jolanta Dadonienė, Dalia Miltinienė, Justinas Ivaška

**Affiliations:** 1 State Research Institute Centre for Innovative Medicine, Santariškių str. 5, LT-08406 Vilnius, Lithuania; tatjana.ivaskiene@imcentras.lt (T.I.); diana.mieliauskaite@imcentras.lt (D.M.); jolanta.dadoniene@mf.vu.lt (J.D.); dalia.miltiniene@santa.lt (D.M.); 2 Clinic of Ear, Nose, Throat and Eye Diseases, Institute of Clinical Medicine, Vilnius University Faculty of Medicine, M.K. Čiurlionio str. 21, LT-03101 Vilnius, Lithuania; eugenijus.lesinskas@santa.lt (E.L.); justinas.ivaska@santa.lt (J.I.); 3 Department of Public Health, Institute of Health Sciences, Vilnius University Faculty of Medicine, M.K. Čiurlionio str. 21, LT-03101 Vilnius, Lithuania; 4 Clinic of Rheumatology, Orthopedics Traumatology and Reconstructive Surgery, Institute of Clinical Medicine, Vilnius University Faculty of Medicine, M.K. Čiurlionio str. 21, LT-03101 Vilnius, Lithuania

**Keywords:** vasculitis, autoimmune disease, hearing loss, audiometry

## Abstract

*Background and Objectives*: Systemic small-vessel vasculitis (SV) represents a group of rare autoimmune disorders with varied etiologies and clinical manifestations. Audiovestibular involvement in SV may present with a broad spectrum of symptoms, often complicating diagnosis and management. This study aimed to evaluate auditory function and speech perception in individuals diagnosed with SV and to investigate associations with disease-specific clinical parameters. *Materials and Methods*: A total of 40 patients diagnosed with SV (mean age: 48.9 years; range: 28–65 years) were recruited for comprehensive audiological assessment. The evaluation protocol included otoscopic examination, tympanometry, pure-tone audiometry, and speech audiometry. Statistical analysis was conducted using R software (version 4.3.1), and significance was set at *p* < 0.05. *Results:* Diagnoses included granulomatosis with polyangiitis (52.5%), eosinophilic granulomatosis with polyangiitis (27.5%), necrotizing vasculopathy (12.5%), and microscopic polyangiitis (7.5%). Mean disease duration was 4.14 years. Hearing complaints were reported by 77.5%; in 20%, they were the initial symptoms. Audiometry identified hearing loss in 50% of patients—predominantly sensorineural (33.8%), followed by mixed (13.7%) and conductive (2.5%) types. Hearing loss was most frequent in necrotizing vasculopathy (60%) and among ANCA-positive individuals (53.7%). *Conclusions*: Sensorineural hearing loss is common in SV, particularly in ANCA-positive patients, highlighting the need for routine hearing assessment in SV management.

## 1. Introduction

Hearing loss is one of the most prevalent health disorders worldwide. According to estimates published by the World Health Organization in 2021, approximately 466 million people—accounting for around 7% of the global population—are affected by hearing impairment [1]. Hearing impairment significantly compromises quality of life by disrupting routine communication, fostering social withdrawal, and contributing to psychological and cognitive dysfunction [2,3]. Affected individuals have been shown to experience higher rates of depression, anxiety, irritability, and diminished self-esteem compared to the general population [4].

There is increasing evidence that hearing loss of varying severity frequently occurs in the context of systemic autoimmune diseases, often alongside other disease-specific clinical manifestations [5,6,7,8,9,10,11,12]. Systemic vasculitis (SV) represents a heterogeneous group of autoimmune rheumatic disorders characterized by inflammation of blood vessel walls [13]. This pathological process is often accompanied by necrosis and occlusive vascular changes, leading to tissue ischemia and necrosis in affected organs. Vascular wall damage, luminal narrowing, and thrombosis are central to the disease pathophysiology [14,15]. The clinical presentation of vasculitis is highly variable and depends on the size and distribution of the affected vessels, the extent of organ involvement, and the degree of circulatory impairment [16,17]. Initially, the disease may manifest in a single organ but often progresses to involve multiple systems. Vasculitides are classified as either primary (idiopathic) or secondary, the latter arising in association with other diseases [18]. Although SV diseases are rare, they are more frequently diagnosed in adults than in children [13]. In Lithuania, the estimated prevalence is approximately 46.9 cases per million inhabitants [19]. Reliable epidemiological data are limited due to the disease’s variable clinical course and frequent relapses.

Diagnosing SV is often challenging due to the lack of specific laboratory biomarkers and the broad spectrum of clinical presentations [20]. Histopathological analysis remains a key diagnostic tool. The classical histological triad—perivascular neutrophilic infiltration, vascular wall necrosis, and intraluminal thrombosis—may support the diagnosis, although it is not universally present [14]. Auditory involvement is a frequently under-recognized manifestation in patients with SV. Clinical presentations may include hearing loss, tinnitus, and vertigo [21,22]. The degree of hearing impairment varies from mild to profound and may be conductive, sensorineural, or mixed in type [23]. The aim of this study was to evaluate auditory function and speech perception in individuals diagnosed with small-vessel SV and to analyze correlations with disease-specific clinical parameters.

## 2. Materials and Methods

This study was carried out between 2022 and 2024 at the State Research Institute Centre for Innovative Medicine, in collaboration with the Department of Ear, Nose, and Throat Diseases at Vilnius University Hospital Santaros Klinikos. Ethical approval was obtained from the Vilnius Regional Biomedical Research Ethics Committee (approval No. 2022/12–1482–948 and date of approval 20 December 2022).

A total of 40 patients diagnosed with small-vessel SV were included in this study. To minimize confounding effects related to age-related auditory decline (presbycusis), individuals older than 65 years were excluded from the study population. The diagnosis of systemic vasculitis was verified by the Department of Rheumatology, Vilnius University Hospital Santaros Klinikos. Audiological assessments were conducted at the Department of Otorhinolaryngology within the same institution. All participants were native Lithuanian speakers.

Each participant underwent a thorough visual examination of the external auditory canal and tympanic membrane using an otoscope, assessing for structural abnormalities and signs of infection. Tympanometry was conducted using the Tymp 4000 impedance audiometer (Homoth, Kaltenkirchen, Germany) with a 226 Hz probe tone at 85 dB. Air pressure in the ear canal was changed between +200 and –400 daPa, and tympanogram results were classified according to the system proposed by Jerger (1970) [24] into three types based on peak height and location: Type A—a peaked curve within –100 to +50 daPa with amplitude >0.2 cm^3^; Type B—a flat curve with no discernible peak; Type C—a peaked curve at <−100 daPa with amplitude >0.2 cm^3^ [24].

Audiometric testing was carried out using a clinical audiometer AC40 (Interacoustics, Assens, Denmark), calibrated to ANSI S3.6–1996 standards, in a sound-treated booth (audiometric cabin, model SAD–2000, Sontek, Rochester, NH, USA). Pure-tone audiometry was used to determine hearing thresholds at 125, 250, 500, 1000, 2000, 4000, and 8000 Hz for both air and bone conduction in each ear. According to the outcomes of pure tone audiometry, hearing loss was categorized into three types: conductive, sensorineural, and mixed. Conductive hearing loss (CHL) was characterized by an air–bone gap greater than 10 dB. Sensorineural hearing loss (SNHL) was defined when both air and bone conduction thresholds exceeded 25 dB, with an air–bone gap of ≤10 dB. Mixed hearing loss (MHL) was identified as the presence of both conductive and sensorineural components [25].

Speech audiometry was employed to evaluate the capacity of speech perception. Testing was conducted using Lithuanian language speech materials, including lists of bisyllabic spondaic and phonemically balanced words [26]. Speech stimuli were played from a Panasonic DVD–S42 (Panasonic, Osaka, Japan) CD player, routed through the audiometer, and delivered via TDH–39 headphones. The speech detection threshold (SDT) was defined as the lowest intensity level at which speech was detected. The speech recognition threshold (SRT) was measured using an ascending method (Downs and Minard, 1996, Baltimore, MD, USA), where words were presented starting at 30 dB below the SDT and increased until 50% of the words were correctly repeated [27].

Word recognition scores (WRSs) were obtained at an intensity level approximately 30 dB above the SRT, using a 25-word list of phonetically balanced stimuli presented in quiet conditions. WRS was expressed as the percentage of words correctly identified by the participant. The speech discomfort level (SDL) was determined by identifying the intensity at which participants reported speech as uncomfortably loud. The dynamic range of speech hearing was calculated as the difference between the SDL and SDT.

Hearing and speech perception outcomes were analyzed in relation to the duration of SV, with participants categorized into three groups: (1) disease duration <1 year; (2) disease duration between 1 and 5 years; and (3) disease duration >5 years. Additionally, to assess the impact of pharmacological treatment on audiological outcomes, participants were stratified based on their therapeutic regimen into the following groups: (1) treated with glucocorticoids only; (2) treated with a combination of glucocorticoids and immunosuppressants; and (3) treated with glucocorticoids, immunosuppressants, and biological agents.

Statistical analysis was performed using R software (version 4.3.1, released 16 June 2023) with the Rcmdr package (version 2.9–1). Continuous data were reported as medians (Q1, Q3, minimum, maximum) and means (standard deviation). The Mann–Whitney U test (Wilcoxon) and Kruskal–Wallis test were used to compare medians between two or three independent groups. Group differences in categorical variables were assessed using Fisher’s exact test. A *p*-value of < 0.05 was considered statistically significant.

## 3. Results

A total of 40 patients diagnosed with small-vessel SV were enrolled in this study. The age of study participants ranged from 28 to 65 years, with a mean age of 48.9 years. The cohort consisted of 21 women (52.5%) and 19 men (47.5%). Detailed demographic and clinical characteristics are presented in Table 1.

The diagnosis was histologically confirmed via biopsy in 97.5% of cases. Anti-neutrophil cytoplasmic antibodies (ANCAs) were assessed using indirect immunofluorescence, with positive findings in 67.5% of patients. The mean duration of autoimmune disease was 4.14 years (range: 0.5–24 years), with a median of 2.25 years.

Auditory symptoms were reported by 77.5% of patients with small-vessel SV. The most frequently reported complaints included hearing loss (52.5%), ear fullness (40.0%), tinnitus (32.5%), and a history of chronic ear infections (17.5%). Otalgia and hyperacusis were each reported by 7.5% of participants. In total, 22.5% reported no auditory complaints.

Review of medical history revealed that in 20.0% of patients, otologic symptoms were the initial clinical manifestation of systemic vasculitis. Among these patients, hearing deterioration was reported as the first symptom in 87.5%, followed by tinnitus (62.5%), ear fullness (37.5%), and otalgia (25%). All patients whose disease began with otologic symptoms were diagnosed with GPA.

Otoscopy findings were normal in 82.5% of participants. Observed abnormalities included exostoses, retracted or immobile tympanic membranes, and postoperative changes or the presence of ventilation tubes. Tympanometry revealed type A tympanograms in 85% of cases. Pure-tone audiometry indicated hearing loss in 50% of patients (Table 2).

**Figure 1:**
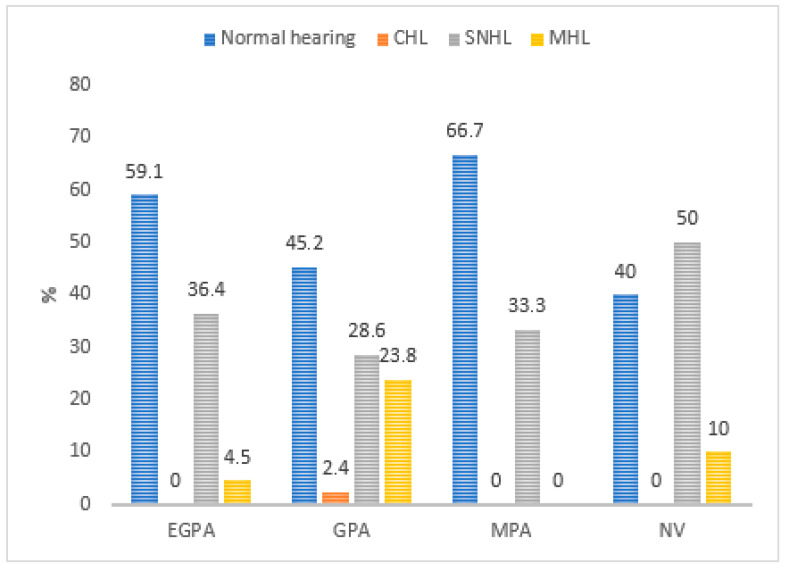
Types of hearing impairment in systemic small-vessel vasculitis subtypes (SNHL—sensorineural hearing loss; CHL—conductive hearing loss; MHL—mixed hearing loss; EGPA—eosinophilic granulomatosis with polyangiitis; GPA—granulomatosis with polyangiitis; MPA—microscopic polyangiitis; NV—necrotizing vasculopathy).

Audiometric findings were further analyzed according to disease subtype, ANCA status, disease duration, and treatment. Hearing loss was most frequent in the NV group, with 60% of cases affected (50% SNHL, 10% mixed). SNHL was also the most common type of hearing loss in the remaining subgroups: 36.4% in EGPA, 33.3% in MPA, and 28.6% in GPA. Conductive hearing loss was identified exclusively in the GPA group (2.4%), which also had a higher incidence of mixed hearing loss (Figure 1).

To further evaluate auditory function across subtypes of SV, pure-tone audiometry thresholds were compared at frequencies from 125 Hz to 8000 Hz. The results, summarized in Table 3, indicate that while there were variations in mean hearing thresholds among the EGPA, GPA, MPA, and NV groups, and these differences did not reach statistical significance across any of the tested frequencies.

In addition to pure-tone audiometry, speech audiometry outcomes were evaluated to assess functional hearing performance. As presented in Table 4, no statistically significant differences were identified among the disease subtypes across any of the speech audiometry parameters.

In the ANCA-positive group, hearing impairment was identified in 53.7% of patients. SNHL was the predominant type of hearing loss in both the ANCA-positive (37.0%) and ANCA-negative groups (26.9%), with a higher prevalence in the ANCA-positive group. Although hearing thresholds at 4000 Hz were slightly higher in ANCA-positive individuals, this difference did not reach statistical significance (*p* > 0.05). Speech audiometry results also showed no statistically significant differences between ANCA groups (Table 5).

**Figure 2:**
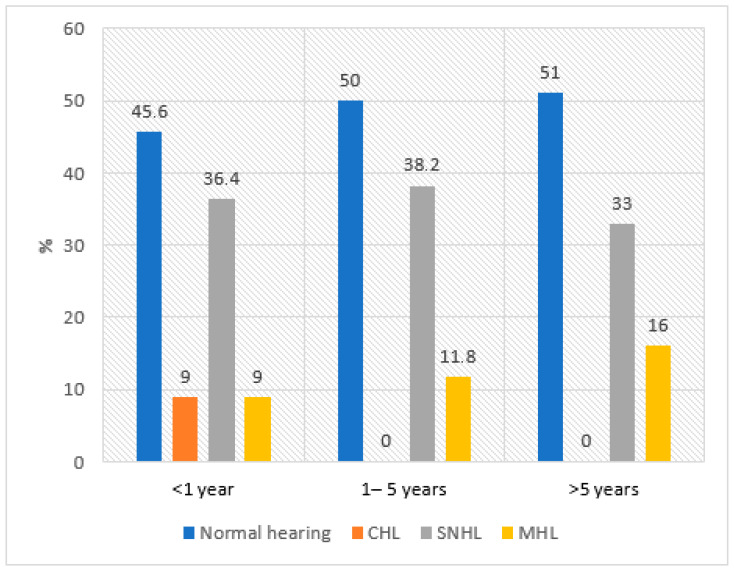
Types of hearing impairment by disease duration in the systemic small-vessel vasculitis group (SNHL—sensorineural hearing loss; CHL—conductive hearing loss; MHL—mixed hearing loss).

Analysis by disease duration showed that SNHL and mixed hearing loss were the most common across all groups. Conductive hearing loss was observed exclusively in the group with disease duration ≤ 1 year (Figure 2).

No statistically significant differences were found in either pure-tone or speech audiometry results among groups stratified by disease duration (Table 6).

When comparing treatment groups, pure-tone audiometry thresholds were slightly better in patients treated with glucocorticoids alone, compared to those receiving combination therapy with immunosuppressants and/or biologics. However, we found no significant difference in pure tone audiometric thresholds between patients treated with glucocorticoids alone and those treated with combination therapy with immunosuppressants and/or biologics (Table 7).

Similarly, no significant differences were observed across treatment groups for speech audiometry parameters, including SDT, SRT, WRS, SDL, and DR.

## 4. Discussion

Hearing and speech perception disorders associated with SV and other autoimmune diseases are becoming an increasingly addressed topic in the current scientific literature [5,6,28,29]. In patients affected by these conditions, hearing loss is the most frequently reported audiovestibular symptom, often accompanied by tinnitus or vertigo [30]. These symptoms, resulting from the autoimmune etiology of the disease and related damage to the middle or inner ear, may be the earliest indicators of an otherwise asymptomatic autoimmune disorder. Conversely, otorhinolaryngologic manifestations may remain unnoticed in patients already diagnosed with a systemic autoimmune disease [31].

Recent studies indicate that hearing disorders are common in individuals with SV. The primary mechanism of impairment is sensorineural hearing loss, although conductive or mixed types of hearing loss can also occur due to fluid accumulation in the middle ear, Eustachian tube dysfunction, or damage to the ossicular chain [32,33,34]. Diagnosing these disorders is often complicated by the variable dynamics of symptoms, ranging from sudden to progressive onset, as well as their severity, which may vary from mild to profound. Moreover, hearing impairments in autoimmune diseases may mimic those caused by other comorbidities such as arterial hypertension, diabetes mellitus, or age-related changes [35].

To avoid confounding due to age-related hearing loss, we excluded individuals over 65 years of age from our study, as epidemiological data suggest that up to 60% of individuals in this age group experience hearing impairment [36,37]. Given that hearing loss in this age range is a normal physiological process, separating age-related changes from those caused by SV would be challenging. By excluding older participants, our study provides a clearer assessment of the impact of SV on hearing and reduces the risk of age-related bias.

Hearing impairments may occur in the context of various autoimmune diseases, with variable prevalence rates depending on the population and study methodology [5,6]. The diagnostic approach to hearing loss in autoimmune diseases includes a thorough medical history, clinical evaluation, laboratory testing, and detailed audiological assessments. Otological symptoms tend to be nonspecific, ranging from signs of mild otitis media to significant hearing loss. These may occur unilaterally or bilaterally and present with either gradual onset or sudden hearing decline. Reported symptoms include hearing deterioration, tinnitus, ear fullness, and otalgia. Importantly, hearing loss may occur at any point during the disease course and may even be the initial sign of an underlying autoimmune disorder [38,39]. In our study, we found that in 20% of cases, the first clinical manifestations of autoimmune disease were related to auditory system dysfunction. Among these patients, hearing loss was the most frequently reported symptom, observed in 87.5% of cases. Notably, all patients whose disease onset involved otologic symptoms were diagnosed with GPA. The literature suggests that in up to 20% of GPA cases, the initial symptoms involve ear pathology, typically presenting as hearing loss, tinnitus, or ear fullness [9,40,41]. Our findings indicate an even higher rate: 38% of GPA patients had otologic symptoms as the first manifestation.

Otoscopy revealed various external and middle ear abnormalities in 17.5% of participants, while type B tympanograms—indicative of middle ear involvement—were observed in 11.3% of cases. Overall, 77.5% of participants with systemic vasculitis reported auditory symptoms, including hearing loss, ear fullness, tinnitus, otalgia, and hyperacusis. These findings are consistent with other studies, which report hearing loss in 20–93% of systemic vasculitis patients, depending on disease subtype and study methodology [14,42].

The literature emphasizes that the most common type of hearing impairment in SV is sensorineural, although mixed and conductive types are also described [6,21]. Our data support these findings: pure-tone audiometry revealed SNHL in 33.8% of participants, while mixed and conductive hearing loss were less frequent. The pathophysiological mechanisms behind these findings likely involve small vessel vasculitis leading to impaired blood flow and inflammation within inner ear structures, resulting in cochlear and auditory nerve damage [7,11]. Conductive hearing loss, particularly in GPA, may result from granulomatous masses impeding ossicular chain movement [43,44,45].

Hearing outcomes differed between vasculitis subtypes, likely reflecting differences in underlying disease mechanisms. Sensorineural hearing loss was most common across all subgroups: 50% in NV, 36.4% in EGPA, 33.3% in MPA, and 28.6% in GPA. Conductive hearing loss was observed exclusively in GPA, which also had a higher incidence of mixed hearing loss. These findings are in agreement with previous studies. For instance, Wali et al. reported hearing loss in 35% of GPA patients [46], while Bakthavachalam et al. found a 56% prevalence, with 47% being sensorineural [47]. The higher rates in those studies may be influenced by participant age, as they included individuals over 65. A 2016 study by Seccia et al. reported hearing loss in 52.8% of EGPA patients, predominantly SNHL [48]. Our findings are comparable, with 41% of EGPA participants experiencing hearing impairment, most commonly of the sensorineural type.

Autoimmune processes in the inner ear, including damage to cochlear hair cells and the spiral ganglion, can also impact speech perception. However, there is limited research on speech audiometry in patients with SV. A 2017 study by Rahne et al. involving 58 patients with autoimmune diseases (22 with rheumatoid arthritis (RA), 20 with systemic lupus erythematosus (SLE), and 16 with GPA) found that speech perception was significantly worse in GPA patients compared to those with RA or SLE [22]. Our study demonstrated that speech perception in individuals with systemic vasculitis fell within the normative range across all groups. However, in the GPA group, the performance (93.9%) was slightly lower compared to the EGPA (96.0%), MPA (100%), and NV (97.9%) groups.

ANCAs, especially prevalent in GPA and MPA, are central to SV pathogenesis. Recent evidence suggests an association between ANCA titers and hearing dysfunction [49,50]. These antibodies mediate neutrophil activation, promoting vascular inflammation and inner ear damage. ANCA-associated mechanisms may also contribute to Eustachian tube dysfunction and otitis media, thereby causing conductive hearing loss. In our cohort, 67.5% of patients were ANCA-positive, and hearing impairment was more common in this group (53.7%) than in ANCA-negative individuals (42.3%).

The severity and type of hearing and speech perception impairment in SV may depend on disease duration, due to cumulative vascular and auditory damage from prolonged inflammation. In early stages, transient conductive hearing loss may be more frequent, often due to reversible factors such as Eustachian tube dysfunction or otitis media [51]. Our findings revealed that conductive hearing loss was observed only in participants with disease duration under one year, while the incidence of sensorineural and mixed hearing loss remained similar across disease duration groups.

Pharmacological treatment of SV—commonly involving glucocorticoids, immunosuppressants, and biologics—can exert both protective and adverse effects on hearing and speech perception. The outcome depends on their anti-inflammatory efficacy versus potential toxicity [52,53]. Our analysis showed that patients receiving only glucocorticoids had slightly better hearing thresholds than those undergoing combination therapy, although these differences were not statistically significant for either hearing or speech perception outcomes.

This study is limited primarily by its modest sample size, due to the rarity of small-vessel SV. Accordingly, subgroup analyses—particularly for MPA—should be interpreted with caution, as the limited number of cases may not adequately represent broader patient populations or disease variability. We also emphasize the need for further investigations involving larger cohorts to more accurately evaluate potential associations with disease subtype, disease duration and treatment modalities, as well as to address potential confounding factors, including comorbidities and the use of ototoxic medications, in order to draw more definitive conclusions regarding these relationships.

## 5. Conclusions

Hearing impairment was identified in 50% of patients with small-vessel SV, with sensorineural hearing loss emerging as the predominant subtype. A higher prevalence of auditory dysfunction was observed among individuals who tested positive for ANCA. Due to the high prevalence of audiological symptoms in this patient population, routine otorhinolaryngologic evaluation and comprehensive hearing assessment should be considered standard components of care for individuals diagnosed with small-vessel SV.

## Figures and Tables

**Table 1:** Demographic and clinical characteristics of study participants.

		Systemic Vasculitis (*n* = 40 Participants)
**Age, Average ±SD**		48.9 ± 11.47
Sex	Female	21 (52.5%)
	Male	19 (47.5%)
Systemic vasculitis disease	Granulomatosis with polyangiitis (GPA)	21 (52.5%)
	Eosinophilic granulomatosis with polyangiitis (EGPA)	11 (27.5%)
	Necrotizing vasculopathy (NV)	5 (12.5%)
	Microscopic polyangiitis (MPA)	3 (7.5%)
Disease duration	<1 year	11 (27.5%)
	1–5 years	17 (42.5%)
	>5 years	12 (30.0%)
ANCA	Positive	27 (67.5%)
	Negative	13 (32.5%)
Medication usage (ever)	Glucocorticoids	9 (22.5%)
	Glucocorticoids and immunosuppressants	20 (50.0%)
	Glucocorticoids, immunosuppressants and biologicals	11 (27.5%)
Organ involvement (ever)	Lung	30 (75.0%)
	Ear, nose, throat	29 (72.0%)
	Kidney	13 (32.5%)
	Nervous system	8 (20.0%)
	Skin	4 (10.0%)
	Musculoskeletal system	3 (7.5%)
	Eye	1 (2.5%)

**Table 2:** Otoscopy, tympanometry, and pure-tone audiometry results in patients with systemic small-vessel vasculitis.

Otoscopy	Normal findings	82.5%
	Exostoses	6.6%
	Retracted tympanic membrane	3.8%
	Immobile tympanic membrane	1.3%
	Postoperative changes in tympanic membrane	1.3%
	Presence of ventilation tube	1.3%
Tympanometry	A type	85.0%
	B type	11.3%
	C type	0.0%
	Not recorded	3.7%
Pure tone audiometry	Normal hearing	50.0%
	Sensorineural hearing loss	33.8%
	Mixed hearing loss	13.7%
	Conductive hearing loss	2.5%

**Table 3:** Distribution of pure-tone audiometric hearing thresholds at 125, 250, 500, 1000, 2000, 4000, and 8000 Hz in subjects with systemic small-vessel vasculitis subtypes (SD—standard deviation).

Frequency	EGPA Group (dB) (*n* = 22 Ears)	GPA Group (dB) (*n* = 42 Ears)	MPA Group (dB) (*n* = 6 Ears)	NV Group (dB) (*n* = 10 Ears)	
	Mean ± SD	Mean ± SD	Mean ± SD	Mean ± SD	*p* Value
125 Hz	13.4 ±12.27	20.2 ± 14.27	5.0 ± 0.00	12.0 ± 5.70	0.055
250 Hz	14.5 ± 14.74	20.5 ± 16.42	5.0 ± 0.00	10.0 ± 3.54	0.060
500 Hz	15.5 ± 17.39	24.5 ± 19.74	11.7 ± 7.64	10.0 ± 3.54	0.156
1000 Hz	15.0 ± 14.83	25.2 ± 21.48	16.7 ± 7.64	15.0 ± 7.91	0.360
2000 Hz	20.2 ± 17.36	26.2 ± 21.56	11.7 ± 2.89	25.0 ± 14.58	0.641
4000 Hz	29.1 ± 23.11	34.8 ± 24.00	16.7 ± 12.58	34.0 ± 21.62	0.537
8000 Hz	35.9 ± 30.81	37.1 ± 30.68	28.3 ± 15.28	45.0 ± 28.28	0.795

**Table 4:** Speech audiometry results in subjects with systemic small-vessel vasculitis subtypes (SD—standard deviation; SDT—speech detection threshold; SRT—speech recognition threshold; WRS—word recognition score; SDL—speech discomfort level; DR—dynamic range of speech).

	EGPA Group (dB) (*n* = 22 Ears)	GPA Group (dB) (*n* = 42 Ears)	MPA Group (dB) (*n* = 6 Ears)	NV Group (dB) (*n* = 10 Ears)	
	Mean ± SD	Mean ± SD	Mean ± SD	Mean ± SD	*p* Value
SDT (dB)	21.8 ±16.92	31.9 ± 22.05	15.0 ± 13.23	25.0 ± 11.18	0.352
SRT (dB)	35.9 ± 21.66	42.4 ± 21.19	25.0 ± 13.23	32.0 ± 10.37	0.400
WRS (%)	96.0 ± 10.88	93.9 ± 19.22	100.0 ± 0.00	97.6 ± 3.58	0.524
SDL (dB)	100.5 ± 6.50	99.5 ± 8.05	106.7± 2.89	97.0 ± 12.55	0.226
DR (dB)	78.6 ± 19.51	67.1 ± 23.64	91.7 ± 10.41	72.0 ± 9.75	0.130

**Table 5:** Pure-tone audiometry and speech audiometry results in ANCA-positive and ANCA-negative systemic small-vessel vasculitis patients (PTA—pure-tone audiometry; SD—standard deviation; SDT—speech detection threshold; SRT—speech recognition threshold; WRS—word recognition score; SDL—speech discomfort level; DR—dynamic range of speech).

	ANCA Positive (*n* = 54 Ears) Mean (±SD)	ANCA Negative (*n* = 26 Ears) Mean (±SD)	*p*- Value
PTA at frequency 500–2000 Hz (dB)	19.4 ± 16.89	22.7 ± 25.83	0.631
PTA at frequency 4000–8000 Hz (dB)	33.7 ± 24.45	34.2 ± 30.0)	0.65
SDT (dB)	25.6 ± 18.19	26.7 ± 23.15	0.832
SRT (dB)	36.5 ± 17.69	38.1 ± 24.98	0.667
WRS (%)	96.7 ± 12.22	92.9 ± 20.96	0.595
SDL (dB)	100.9 ± 6.94	97.9 ± 9.18	0.199
DR (dB)	75.2 ± 19.45	71.2 ± 25.78	0.581

**Table 6:** Pure-tone audiometry and speech audiometry results in systemic small-vessel vasculitis patients, stratified by disease duration (Group 1—disease duration < 1 year; Group 2—disease duration between 1 and 5 years; Group 3—disease duration > 5 years; SD—standard deviation; SDT—speech detection threshold; SRT—speech recognition threshold; WRS—word recognition score; SDL—speech discomfort level; DR—dynamic range of speech).

	Group 1 (*n* = 22 Ears) Mean (±SD)	Group 2 (*n* = 34 Ears) Mean (±SD)	Group 3 (*n* = 24 Ears) Mean (±SD)	*p*- Value
PTA at frequency 500–2000 Hz (dB)	22.2 ± 25.72	18.5 ± 16.81	21.6 ±19.28	0.944
PTA at frequency 4000–8000 Hz (dB)	36.5 ± 31.13	31.8 ± 23.39	34.3 ± 25.95	0.986
SDT (dB)	28.2 ± 21.80	25.6 ± 17.79	24.4 ± 21.23	0.567
SRT (dB)	39.3 ± 20.55	36.0 ± 17.05	36.3 ± 24.33	0.438
WRS (%)	89.0 ± 27.80	98.4 ± 2.97	97.3 ± 7.70	0.627
SDL (dB)	97.5 ± 9.85	100.6 ± 8.14	101.3 ± 4.23	0.568
DR (dB)	68.9 ± 23.50	75.0 ± 19.66	76.9 ± 22.64	0.276

**Table 7:** Pure-tone audiometry and speech audiometry results in systemic small-vessel vasculitis patients treated with different medication regimens (Group 1—treated with glucocorticoids only; Group 2—treated with glucocorticoids and immunosuppressants; Group 3—treated with glucocorticoids, immunosuppressants, and biologic therapy; SD—standard deviation; SDT—speech detection threshold; SRT—speech recognition threshold; WRS—word recognition score; SDL—speech discomfort level; DR—dynamic range of speech).

	Group 1 (*n* = 18 Ears) Mean (±SD)	Group 2 (*n* = 40 Ears) Mean (±SD)	Group 3 (*n* = 22 Ears) Mean (±SD)	*p*- Value
PTA at frequency 500–2000 Hz (dB)	18.1 ± 26.89	20.1 ± 15.16	23.0 ± 22.34	0.103
PTA at frequency 4000–8000 Hz (dB)	31.9 ± 31.68)	34.5 ± 23.22	34.2 ± 27.62	0.541
SDT (dB)	22.5 ± 22.44	28.4 ± 15.99	24.3 ± 23.82	0.054
SRT (dB)	33.1 ± 20.30	39.5 ± 18.36	35.7 ± 23.42	0.110
WRS (%)	93.6 ± 23.45	96.6 ± 7.44	95.0 ± 18.93	0.477
SDL (dB)	98.3 ± 9.70	99.3 ± 8.29	102.5 ± 4.01	0.193
DR (dB)	75.8 ± 23.34	70.6 ± 19.29	78.2 ± 24.18	0.133

## Data Availability

The dataset collected and/or analyzed in the present study is available upon request from the corresponding author.
